# Barriers and facilitators for implementation of the SWORD evidence-based psychological intervention for fear of cancer recurrence in three different healthcare settings

**DOI:** 10.1007/s11764-022-01285-x

**Published:** 2022-11-04

**Authors:** Esther Deuning-Smit, Evie E. M. Kolsteren, Linda Kwakkenbos, José A. E. Custers, Rosella P. M. G. Hermens, Judith B. Prins

**Affiliations:** 1 Department of Medical Psychology, Radboud Institute for Health Sciences, Radboud University Medical Center, PO Box 9101, Geert Grooteplein Zuid 10, Nijmegen, HB 6500 the Netherlands; 2 Department of Clinical Psychology, Radboud University, Nijmegen, The Netherlands; 3 Department of IQ Healthcare, Radboud Institute for Health Sciences, Radboud University Medical Center, Nijmegen, the Netherlands

**Keywords:** Fear of cancer recurrence, Cancer survivors, Psychological interventions, eHealth, Implementation, Barriers

## Abstract

**Purpose:**

Fear of cancer recurrence (FCR) interventions are effective, but few are implemented. This study aimed to identify barriers and facilitators for implementing the evidence-based blended SWORD intervention in routine psycho-oncological care.

**Methods:**

Semi-structured interviews with 19 cancer survivors and 18 professionals from three healthcare settings assessed barriers and facilitators in six domains as described by the determinant frameworks of Grol and Flottorp: (1) innovation, (2) professionals, (3) patients, (4) social context, (5) organization, and (6) economic and political context.

**Results:**

In the *innovation domain*, there were few barriers. Facilitators included high reliability, accessibility, and relevance of SWORD*.* In the *professional domain*, physicians and nurses barriers were lack of self-efficacy, knowledge, and skills to address FCR whereas psychologists had sufficient knowledge and skills, but some were critical towards protocolized treatments, cognitive behavioral therapy, or eHealth. *Patient domain* barriers included lack of FCR awareness, negative expectations of psychotherapy, and unwillingness/inability to actively engage in treatment. A *social context domain* barrier was poor communication between different healthcare professionals. *Organization domain* barriers included inadequate referral structures to psychological services, limited capacity, and complex legal procedures. *Economic and political context domain* barriers included lack of a national implementation structure for evidence-based psycho-oncological interventions and eHealth platform costs.

**Conclusions:**

Implementation strategies should be targeted at patient, professional, organizational and economic and political domains. Identified barriers and facilitators are relevant to other researchers in psycho-oncology that aim to bridge the research-practice gap.

**Implications for cancer survivors:**

This study contributes to the implementation of evidence-based psychological interventions for cancer survivors, who can benefit from these services.

**Supplementary Information:**

The online version contains supplementary material available at 10.1007/s11764-022-01285-x.

## Introduction

In the last decades, many evidence-based psychological interventions have been developed to support cancer survivors [1]. Unfortunately, the clinical implementation of these interventions falls short [2, 3]. Bridging the gap between evidence and practice has therefore been acknowledged as one of the most important current challenges in psycho-oncology [3, 4] which might be approached with methods used in implementation science [2, 3, 5]. While experimental research tests the efficacy and cost-effectiveness of an intervention in a controlled study context, implementation science aims to understand whether an intervention is (cost-)effective and feasible within a real-world context [2]. Identifying potential barriers and facilitators for implementation is an important first step. Interventions could then be adapted and strategies tailored to these barriers and facilitators developed to support successful implementation.

Fear of cancer recurrence (FCR), as most important unmet need of cancer survivors [6], is an important topic within the psycho-oncology with a prominent gap between intervention research and clinical practice [7]. FCR, defined as “the fear or worry or concern that the cancer will return or progress” [8] ranges from adaptive levels to clinical FCR which is characterized by (1) high levels of worry and (2) high levels of preoccupation that are (3) persistent over time and (4) hypervigilance to bodily symptoms [9]. Clinical FCR is associated with impaired quality of life [10] and increased healthcare use [11] and might require psychological interventions to manage symptoms [1]. Although multiple evidence-based FCR interventions have been developed and tested in randomized controlled trials (RCTs), few have been implemented in daily clinical practice. Evaluating FCR interventions in real-world settings has therefore been identified as one of the highest research priorities on the international FCR research agenda [12].

The Survivors’ Worries of Recurrent Disease (SWORD) intervention is a blended psychological intervention for FCR based on cognitive behavioral therapy (CBT) [13]. The primary emphasis of the therapy is to modify perpetuating factors of high FCR including dysfunctional cognitive thinking patterns (e.g., unhelpful thoughts, rumination) and maladaptive behavior strategies (e.g., avoidance and excessive self-monitoring). SWORD compromises eight sessions with a psychologist accompanied by an interactive eHealth platform with psycho-education and at-home exercises. A more detailed description of SWORD has been published elsewhere [13]. The (long-term) efficacy and cost-effectiveness of SWORD have been confirmed in a randomized controlled trial conducted in an academic hospital setting [14, 15]. To support successful implementation of SWORD in different care settings, it is crucial to understand how the intervention fits in the complex context of psycho-oncological care, and identify potential barriers and facilitators for implementation [16].

Two existing studies have reported barriers and facilitators for FCR-specific interventions. In one study, specialized breast cancer nurses were asked about the challenges they experienced in assessing and managing FCR [17]. Results showed that nurses were open to FCR, but did not always address it in their consultation because they were unclear how to act when patients reported FCR and lacked the tools for adequate assessment. Butow and colleagues examined therapists views regarding sustainable delivery of the evidence-based FCR intervention ConquerFear in routine clinical practice [18]. Important themes were reported in three domains which when present, were facilitators, and if absent, were barriers. In the evidence domain, it was important that the intervention was perceived as credible, efficacious, and necessary. In the context domain, important factors were organizational priority and capacity for addressing FCR, the match between the intervention and therapists orientation and attitude, and sufficient referral from clinical health professionals. In the implementation domain, factors were a good fit between the intervention and the service (e.g., patient flow, session structure), a fit between the intervention and patients’ needs and preferences, and the availability of training and resources. More studies have been published in the broader context of the integration of psycho-oncological care in clinical practice which are summarized in a recently published review [7]. While existing studies provide some useful insights, they predominantly represent views of physicians and nurses and occasionally of psychologists, while views of other relevant stakeholders, including patients and those involved with organizational and contextual aspects of implementation, remain underrepresented. Furthermore, as psycho-oncological care is not limited towards hospitals, it is essential to explore the fit of FCR interventions in different settings, including primary care and mental health settings. This information is crucial to move forwards to broader implementation.

This study aims to assess facilitators and barriers for implementation of SWORD within current psycho-oncological care for cancer survivors with high FCR from different relevant stakeholder perspectives across psycho-oncology settings. The framework of Grol [16] and Flottorp [19] was used to categorize barriers and facilitators for implementation in six domains including characteristics of the (1) innovation, (2) patients, (3) professionals, (4) social context, (5) organization, and (6) economic and political context. This study is the first part of the FORwards project that will evaluate the feasibility of implementation of the SWORD intervention. The findings in this study will be used to adapt SWORD, inform a future feasibility and effectiveness study, and eventually develop a tailored implementation strategy for SWORD. The study results will also inform other researchers within the field of psycho-oncology involved in intervention research.

## Methods

Individual semi-structured interviews were conducted with patients and other relevant stakeholders (psychologists, physicians, nurses, and managers) from three healthcare settings: one academic hospital, one regional hospital, and one psycho-oncological center (POC), in different regions in the Netherlands. These settings were purposefully chosen to represent the type of settings where psychological care for high FCR is provided in the Netherlands. We also conducted interviews with an implementation and healthcare insurance expert outside these settings. The study was reported according to the consolidated criteria for reporting qualitative studies [20]. Ethical approval was obtained by the ethical committee (CMO Arnhem-Nijmegen 2019–5307).

### Participants

Patient participants were outpatients at a medical department or the medical psychology department in the participating hospitals or received psychological therapy at the participating POC. Participants in this study had not been exposed to the SWORD intervention and did not participate in other studies involving SWORD. Eligible patients were (1) diagnosed with cancer at least 6 months prior to inclusion, (2) completed primary medical treatment with a curative intent, and (3) had sufficient command of the Dutch language to comprehend written information and to participate in interviews. Exclusion criteria were terminal/palliative disease stage or being physically unable to participate. We used a purposive case sampling approach to obtain a sample with a maximal variety in cancer type, age, sex, and level of FCR. Physicians, nurse specialists, and psychologists within the three settings identified eligible patients and provided them with written study information. The researcher (ED) monitored whether there was sufficient variation within each category and communicated this with recruiting healthcare providers. The researcher contacted interested patients by phone to provide further information. Written informed consent was obtained by mail.

Professional participants were involved in clinical oncology or psycho-oncological care at one of the participating centers. In both hospitals, relevant stakeholders were psychologists with clinical registration, physicians (oncologist/surgeon), specialized nurses, social workers, and business managers. In the POC, relevant stakeholders were psychologists and business managers. The researcher (ED) contacted a psychologist involved in psycho-oncology care in each center who selected professionals based on a purposive sampling approach to obtain a sample representing all relevant stakeholders. Professionals were contacted to participate in an interview by this psychologist, or directly by one of the researchers. The implementation expert and a healthcare insurance expert outside the participating centers were contacted by the researcher.

Recruitment was performed simultaneously with the data coding and continued until saturation was achieved. The three criteria for saturation were (1) pre-defined desired variation in patients characteristics and professionals background, (2) all topics in the interview guide addressed, and (3) no new themes or subthemes identified through data coding.

### Data collection

Patients received a purpose-designed questionnaire by email prior to the interview, assessing socio-demographic characteristics (age, sex, education), clinical characteristics (cancer type, received cancer treatment, time since diagnosis), and the level of FCR measured by the Cancer Worry Scale-6 (CWS-6). The CWS-6 includes six items rated on a 4-point Likert scale with total scores ranging from 6 to 24 (21). Professionals were asked about their age, professional background, and (when relevant) years of oncology experience.

Interviews were conducted by a researcher (ED) with a master’s degree in psychology and trained in qualitative research that had no prior connection to the patients. Interviews took about 60 min and were audio recorded. Most patient interviews took place face-to-face at the service locations and the last four interviews with patients were conducted by phone as a result of the restrictions due to the COVID-19 pandemic. Professionals were interviewed face-to-face at their work, or by phone or video-call. Two interview guides (one for patients and one for professionals) were developed following the determination framework of Grol [16] and Flottorp [19] for the identification of barriers and facilitators for implementation of care innovations. For each domain in the framework, the interview guides included a general question (e.g., “with regard to the professionals, which barriers/facilitators could we encounter when we implement SWORD?”) and secondary questions addressing the subdomains within the theoretical framework (e.g., “do you expect professionals to have sufficient knowledge and skills to deliver SWORD?”)*.* Open questions were included to assess current care for FCR. The interview guides were adapted when results from initial coding of interviews suggested that certain themes needed deeper understanding. Translated topic guides are included as supplementary file 1.

The interviews were structured as follows: the researcher started to clarify her professional background and role and the plan for analysis and dissemination of the data. Participants were then presented with a short verbal description of SWORD guided by several exemplary exercises and screenshots of the SWORD website on paper. To place the barriers and facilitators into context, we started with asking a description of current care for FCR. Patients were asked about the care they received for FCR, professionals about formal and informal methods they used to screen, refer for and manage FCR, local resources, and regulations. Subsequently, both patients and professionals were asked which barriers and facilitators they expected for the implementation of SWORD within each domain of the theoretical framework: innovation, patients, professionals, social context, organization, and economic and political context. Interviews ended with a general question about possible barriers and facilitators that have not been addressed in one of the domains. The last few interviews of both groups were used to verify findings and cover topics that remained underrepresented.

### Data analysis

Audio-recordings were transcribed verbatim. Using the qualitative software package Atlas.ti Version 9. Approximately one-third of the transcripts were coded independently by two researchers (ED and EK) to develop the coding tree. The remaining transcripts were coded by one researcher (ED) and reviewed by a second researcher (EK). The coders discussed all codes until consensus was achieved and discussed whether data saturation was achieved. Results were regularly shared with the research team.

Data regarding current FCR care was coded following thematic analysis. Initial codes were generated from the data (open coding) after which codes were clustered, split, and/or reformulated into subthemes and higher order themes to form an index which was applied to the additional data. Coding of the barriers and facilitators followed a deductive framework approach. Codes were structured according to the main domains and subthemes of the theoretical framework of Grol [16] and Flottorp [19]. When barriers or facilitators were identified that did not fit with the framework, new subthemes were added to the coding system. After coding was completed, data was charted according to the thematic framework and interpreted by looking for explanations within literature, clinical practice, and the data itself. When needed, codes and subthemes were clustered and/or reformulated to obtain a comprehensive overview of barriers and facilitators.

## Results

### Background information of the participants

Saturation of data was achieved after 19 patient and 18 professional interviews. During analyses, thirteen (35%) transcripts were double coded. Characteristics of participants are displayed in Table 1. Patients mean age was 48, 74% were female, and patients had a history of breast cancer (*n* = 10), colorectal cancer (*n* = 2), renal cancer (*n* = 1), vulvar cancer (*n* = 1), leukemia (*n* = 1), Hodgkin lymphoma (*n* = 1), central nervous system lymphoma (*n* = 1), Ewing sarcoma (*n* = 1), or melanoma (*n* = 1). Professionals had a mean age of 47.7 and 61% was female. A brief description of current care for FCR in the participating healthcare settings is outlined in Box 1.

**Table 1:** Characteristics of the participants

*Patients (n* = *19)*	Mean (range; SD)	*n* (%)
Center		
Academic hospital General hospital Psycho-oncological center		6 (31.6) 6 (31.6) 7 (36.8)
Age	48.00 (25–73; 3.15)	
Female		14 (73.7)
Tumor type		
Breast Colorectal Other		10 (52.6) 2 (10.5) 7 (36.8)
Time since diagnosis (months)	24.05 (6–59; 2.87)	
Treatment		
Surgery Radiotherapy Chemotherapy Hormone therapy Other		3 (15.8) 8 (42.1) 6 (31.6) 13 (68.4) 4 (21.1)
Cancer Worry Scale-6	12.21 (6–20; 3.54)	
***Professionals (n* = *18)***		
Center		
Academic hospital General hospital Psycho-oncological center		6 (33.3) 6 (33.3) 4 (22.2)
Age	47.72 (29–64; 9.22)	
Female		11 (61.1)
Profession		
Psychologist Nurse specialist Physician Social worker Manager Experts		6 (33.3) 4 (22.2) 2 (11.1) 1 (5.6) 3 (16.7) 2 (11.1)
Working experience in oncology (years)	11.94 (0–37; 9.53)	

Box 1 Description of current care for high FCR in three settings in the Netherlands.

**Table:** 

**Providers**: Psychologists that treated FCR in both hospitals had a clinical registration. In the POC, only complex cases were treated by psychologists with clinical registration and other patients by psychologist with a graduate degree. In the POC, all therapists only treated patients with cancer; in the academic hospital, several dedicated psychologists mainly treated patients with cancer; and in the regional hospital, all psychologists treated patients with different medical diagnoses
**Screening and referral**: In both hospitals, patients were referred by physicians or nurse specialists based on clinical insight and in few cases based on a high score on a general distress screening instrument. In the POC, patients were referred by their general practitioner or by their physician or nurse within the hospital
**Nature of FCR care**: Both hospitals offered a multimodal oncological rehabilitation program including psychological (group) sessions covering FCR. Patients with clinically significant FCR were offered individual psychological treatment directed at FCR, either directly or after the rehabilitation program. The POC only offered stand-alone treatment. Treatments were predominantly individual rather than in a group. Neither institution offered a treatment specific for FCR. Most psychologists did not work with treatment protocols but used (elements of) cognitive behavioral therapy, acceptance and commitment therapy, mindfulness, and meaning-centered therapy
**eHealth**: The academic and general hospitals were in the orientation phase towards working with eHealth while in the POC eHealth was well established
**Financing**: Psycho-oncological care for FCR was covered via the basic health insurance that is mandatory for all residents in the Netherlands according to the Dutch health insurance law. Treatment was covered via medical reimbursement (hospitals) or mental healthcare reimbursement (all settings). Mental health reimbursement requires a formal diagnosis of anxiety disorder and the number of sessions covered is based on the severity of FCR

### Barriers and facilitators for implementation of FCR interventions

The semi-structured interviews identified a wide variety of barriers and facilitators in the six domains which are outlined in Table 2. Differences and similarities in reported results between patients and professionals are marked in the table. Here, we summarize the integrated results of the perspectives from patients and professionals from three settings.

**Table 2:** Barriers and facilitators for implementation of SWORD

**Barriers and facilitators of the innovation**		
**Subtheme**	**Barriers**	**Facilitators**
Cultural appropriateness		----eHealth fits with digitalization in society and healthcare ----Psychological health is recognized within oncological care
Credibility		----The content of SWORD is recognizable ----SWORD is evidence-based ----SWORD is cost-effective** ----SWORD is provided by an acknowledged eHealth platform**
Accessibility	----Accessing the eHealth environment requires extra effort for patients* ----eHealth environment is not compatible with existing digital healthcare systems**	----A paper version of SWORD is available ----Patients can complete assignments at their own pace
Applicability	----The content requires timely updates**	----The content matches existing practice ----The structure fits within existing practice
Advantages/ Relevance	----Overlap with existing eHealth psychological interventions	----FCR is a significant problem ----No evidence-based FCR intervention is available** ----eHealth improves information transfer** ----Protocolized care is efficient** ----Online contact is more efficient than face to face contact**
**Barriers and facilitators of the professionals**		
**Subtheme**	**Barriers**	**Facilitators**
*Physicians and nurses (referrers to SWORD)*^*ǂ*^		
Attitude	----Hesitant to change existing routines ----Focus on physical health ----Having reservations about eHealth** ----Feeling they can treat FCR themselves (PH)**	----Open to new routines** ----Open to psychological health ----Open to eHealth** ----Positive experiences with the psychological intervention
Awareness	----Unaware that their patients might have psychological problems (PH)**	----Awareness of FCR**
Self-efficacy	----Experiencing lack of self-efficacy to address mental health**	
Knowledge/skills	----Experiencing a lack of knowledge and skills to address and screen for FCR** ----Experiencing a lack of knowledge and skills to adequately refer patients to psychological services	----Experiencing sufficient knowledge and skills to address and screen for FCR ----Experiencing sufficient knowledge and skills to adequately refer patients to psychological services
Routines and behavior	----FCR is rarely addressed in follow-up appointments ----Few referrals to psychological services (PH)** ----Limited time to address FCR in consultations	----Some address FCR in follow-up appointments ----Some refer to psychological services ----Sufficient time to address FCR in consultations (N)
*Psychologists (providers of SWORD)*		
Attitude	----Hesitant to change existing routines ----Critical of protocolized treatments ----Having reservations about eHealth** ----Critical of cognitive behavioral therapy**	----Open to new routines** ----Positive about protocolized treatments** ----Positive about eHealth and blended care**
Knowledge/skills	----Experiencing lack of knowledge about treatment indication for SWORD**	----Experiencing sufficient knowledge and skills to use SWORD
Routines and behavior	----Not working with cognitive behavioral therapy** ----Not working with protocolized treatments**	----SWORD fits within current routines**
**Barriers and facilitators of the patients**		
**Subtheme**	**Barriers**	**Facilitators**
Needs/preferences	----Having reservations about eHealth ----Having reservations about protocolized treatments ----Preferring peer contact via a treatment group or forum ----No need for learning about experiences from other patients	----Positive about eHealth* ----Positive about protocolized treatment ----Preferring individual treatment ----Need to learn about experiences from other patients ----Need for recognition of FCR ----Preferring guided treatment over self-management
Awareness	----Not knowing when FCR is problematic* ----Not aware that psychological help for FCR is available*	----Timely awareness of available psychological help for FCR
Knowledge/skills	----Having difficulty asking for help ----Limited digital skills ----Limited reading/writing skills ----Limited mastery of the Dutch language ----Limited ability to comprehend complex thought exercises** ----Difficulty to express and reflect on emotions	----Being outspoken in medical appointments* ----Having sufficient digital skills
Motivation	----Not open to psychological help during or shortly after medical treatment ----Not wanting to be in the “patient” role again* ----Not motivated to actively engage in psychological treatment	----Having a need for help with FCR ----Being motivated to actively engage in psychological treatment ----Seeing treatment progress as featured by SWORD is motivating*
Preconditions	----No time or ability to actively engage in psychological treatment ----Not having a computer* ----Long travel time to the clinic ----Physical problems hinder completing the online assignments	----eHealth reduces travel time
Expectations	----Expectation that mental health should not be discussed in medical consultations ----Negative expectations of psychological help	----Positive expectations of psychological help ----Expectation that SWORD will help reducing FCR
**Barriers and facilitators of the social context**		
**Subtheme**	**Barriers**	**Facilitators**
Culture	----Psychological topics are rarely discussed between medical doctors**	----Importance of psychological care is acknowledged by whole medical team** ----Psychological teams are open to eHealth** ----Psychological teams are open to new routines** ----Professionals feel heard by their managers**
Cooperation	----Lack of communication between different care providers about the care patients receive	----Low threshold communication between referrers and psychologists ----Active feedback from psychologists to referrers ----Hospital collaborates with primary care to offer psychosocial care**
Patient social environment	----Not wanting to burden loved ones with FCR concerns* ----Experiencing lack of understanding about FCR from their social environment ----Afraid that their social environment is critical about psychological help*	----Social environment supports patient seeking psychological help ----Patient is open about FCR to loved ones ----Patient is not influenced by opinions from others about FCR* ----SWORD addresses the role of loved ones in managing FCR
Patient-professional relation		----The patient feels seen by their healthcare provider ----The patient feels a connection with their healthcare provider
**Barriers and facilitators of the organization**		
**Subtheme**	**Barriers**	**Facilitators**
Capacity and resources	----Limited capacity of psychologists ----Small institutions have limited budget to purchase licenses for eHealth platforms**	----Bigger institutions have sufficient budget to purchase licenses for eHealth platforms**
Structures	----Institution is dependent on approval from umbrella organization** ----Institution operates in multiple locations which hinders implementation**	----Psychologists and referrers are united in a center for oncological care**
Organization of care processes	----FCR is no structural topic in oncological follow-up ----Not all tumor types have a structured follow-up** ----The distress thermometer is not sufficiently implemented** ----Referral infrastructure is insufficiently embedded in care processes** ----Nurse specialist does not receive formal education about psychosocial care** ----Organizations have different opinions about the qualification of psychologists that provide FCR care	----The distress thermometer is integrated in follow-up care** ----Multidisciplinary and interdisciplinary consultation is embedded within the organization ----Case managers facilitate the screening of FCR ----Referral infrastructure is embedded in care processes
Capacity for organizational change	----Internal legal procedures are complex** ----Medical healthcare has priority** ----Psychologists have little time to get used to new psychological interventions** ----Organization does not work with eHealth** ----Organization works with different eHealth platforms	----Psychosocial care is regarded important** ----Organization has an active structure for implementation** ----External support with implementation** ----Organization works with eHealth** ----Organization regards eHealth important**
**Barriers and facilitators of the economic and political context**		
**Subtheme**	**Barriers**	**Facilitators**
Finances	----Difficult to find a party that wants to invest in implementing and sustaining SWORD** ----License fees are high due to contracts with multiple commercial eHealth platforms** ----Healthcare insurance does not cover platform fees** ----Psychological care in hospitals bound to medical reimbursement**	----SWORD fits within the current healthcare reimbursement for psycho-oncological care**
Legislation	----eHealth is bound to privacy laws ----It is not clear who owns the rights of the psychological intervention material**	
Policy	----There are different opinions about which location is most suitable to offer psycho-oncological care	
Infrastructure for implementation	----There is no nationwide structure that ensures implementation of evidence-based psychological interventions**	

Results are reported both by patients and professionals unless indicated: *only reported by patients; **only reported by professionals

^ǂ^Barriers and facilitators are applicable to both groups of referrers unless specified by (PH) physician or (N) nurse

#### Characteristics of the innovation

One of the barriers of the innovation (SWORD) pertained to the *accessibility* of the eHealth environment, being not compatible with existing digital healthcare systems. This increases the burden upon psychologists and patients as they have to work with multiple systems and use workarounds to transfer information between systems.

A facilitator of the innovation included its high *credibility*. Both professionals and patients were likely to accept SWORD because of its recognizable content, its proven efficacy in reducing FCR, its cost-effectiveness, and the embedding within an acknowledged eHealth platform.

Patient: There is a clear rationale behind it [SWORD], that provides trust to patients, that you believe it will be allright…. Because it is an approved mehod and there are more patients following this [intervention].

Related to *applicability*, a barrier for continuity of the innovation was the requirement of timely updates of the content in order to follow research developments in the field of FCR. A facilitator was that SWORD was perceived congruent with existing care for FCR in the three settings, which thus requires little extra resources for implementation.

Manager: We actually already do this [SWORD] in part, but now every psychologist is doing this in their own way or on their own initiative. When we will use this program, it is a substitution.

Several facilitators were documented regarding the *relevance* of SWORD. Participants perceived FCR as a significant problem after cancer. Existing psychological interventions and techniques were not specific for FCR and perceived as insufficient when FCR was the primary problem.

Psychologist: I welcome [a program] like this. It is a justification that treating anxiety in patients with cancer is truly different from treating “normal” anxiety.

Another facilitator related to *relevance* was that both the protocolized and blended format of SWORD improved the efficiency of psychological treatment.

#### Characteristics of the professionals

An important universal factor among all professionals was their *attitude* towards new routines. Some were open to new developments, while for others it required more effort to change their routines. Specifically, professionals’ attitude regarding eHealth was identified as a barrier as some expected to need advanced technical skills to work with eHealth, perceived eHealth as inferior to personal contact, or expected eHealth to be a barrier for patients.

##### Physicians and nurses (referrers to SWORD)

Although medical health was physician’s and nurse’s main focus, they had an open *attitude* towards patients’ psychological wellbeing. Most had the *awareness* of potential FCR in their patients and the intention to address this in consultations, acting as facilitators. This was in contrast with what was reported about their actual *behavior*. Physicians in particular rarely asked patients directly about FCR, unless patients brought it up themselves. Some incorporated FCR in their communication implicitly, for example, by offering medical reassurance.

Patient: In retrospect, I wish I had known a bit more about what happens to you afterwards [cancer treatment] so that you are more aware that it is not done after the last day of treatment. To know that there is still a long way to go, physically, but certainly also mentally.

Several barriers were brought up for addressing and referring for FCR. Physicians had limited consultation time in their current *routines* and perceived lack of *knowledge and skills* concerning FCR which was complicated by the wide array of survivorship questions they were confronted with. The most important knowledge gap reported by physicians and nurses was how to distinguish “normal” FCR from clinically relevant FCR and they wished to have more guidance to make an informed decision about referral.

Physician: You always go back to the skills you master, which is of course your own profession. That is easy. You do not feel comfortable if you do not precisely know that information [about FCR].

Compared to physicians, nurses’ *routines* included more regular and longer consultations which facilitated addressing psychological wellbeing. Most nurses also experienced sufficient *knowledge and skills* to talk about FCR.

Nurse: I have to say that particularly the nurses are working very hard to raise awareness for psychosocial care. We [nurses] are well equipped to do this because we are involved in all the clinical care pathways.

The results related to referrers were predominantly mentioned by participants from the hospital settings, as the POC receives referrals from outside the setting. However, patients from the POC also reported that FCR was insufficiently addressed during oncological follow-up care, complicating referral to the POC.

##### Psychologists (providers of SWORD)

Facilitators included that SWORD fitted within the current *routines* and *knowledge and skills* of psychologists. The only knowledge barrier pertained to the treatment indication for SWORD, as some wondered for which patients SWORD would be the best suited treatment.

Psychologist: so many [interventions] have been developed that show overlap. At a certain point you wonder, which guideline, which protocol can I use? They are all evidence-based at a certain point.

Concerning psychologists’ *attitudes* towards the protocolized format of SWORD, some perceived that this would not do right to a patient’s individual needs and would leave little room for creativity and flexibility. Another concern was that a protocol would only work for non-complex or “typical” patients, not representing the majority of patients in their setting. Others had a more positive attitude towards protocolized treatment and perceived them as a guideline, leaving sufficient possibilities for personalization. They also expected that a protocol could bring more focus to treatment sessions.

Psychologist: So, you would have a guideline for your treatment, and you adapt this to the individual person with additional building blocks or extra side paths, where needed to adjust the treatment to the client.

Regarding psychologists’ *attitude* towards the therapeutic orientation of SWORD, some were concerned that it would be too much focused on traditional CBT, which they perceived as being too rational. One psychologist also questioned whether cognitive restructuring would the right strategy as FCR is a realistic fear.

#### Characteristics of the patients

An important facilitator was that in general, SWORD fitted patients’ *needs and preferences*. A treatment specifically developed for FCR felt like a validation of their own concerns. This need for recognition was also reflected in their wish for identification with fellow patients. While some patients preferred a group treatment or online forum, most accepted peer comparison through patient videos as included in SWORD as a valuable alternative.

Patient: The fact that there is such a treatment makes you feel that there is some level of understanding, that you are not crazy.

Patients were in general positive about eHealth and had sufficient digital *skills*. For some patients, eHealth could be a barrier because of limited digital *skills*, *preference* for working on paper, or feeling anonymous when working on a website.

One of the most frequently mentioned barriers among patients was negative *expectations* of psychological help. Needing psychological help was for example associated with “having lost your mind,” “failing,” and “being a softy” and psychological treatment was expected to be “woolly” or “having to put everything on the table.” For some, these expectations led to feelings of shame, hesitation to address FCR in medical consultations, and not being open for referral to psychological help.

Patient: You don’t go to a birthday party and tell others that you are seeing a psychologist. It’s a bit of a taboo subject, that you cannot cope on your own or that you are failing.

Also lack of *awareness* about FCR was a barrier to ask for help. Patients did not know how to distinguish normal from problematic FCR and were not aware of the availability of psychological assistance.

Patient: For a lot of things [psychological consequences], you think it’s all part of the game, and when you’ve been “released” after [cancer] treatment, so to say, it’s harder to come back [to the oncologist/medical team] again.

Another barrier hindering patients in addressing FCR was the *expectation* that medical appointments ought to be solely about medical wellbeing. Patients that are not outspoken, or find it difficult to ask for help, might not mention mental health when their physician or nurse does not address it.

Related to *motivation*, most patients experienced a need for help with FCR and were willing to actively invest in psychological treatment. Reasons for not being motivated included not being convinced of the benefits of treatment or not feeling burdened by FCR. Also the timing of introducing SWORD was crucial, and patients mentioned that it should not be offered during or even directly after medical treatment. They needed time to recover from and process the intense phase of diagnosis and treatment and their first priority was to start up life after cancer, leaving little room for psychological treatment.

Patient: If you want to change something, you should want that for 100%. Not only saying you want that, but actually do it…. [people should be] truly convinced that it [psychological treatment] is usefull, that it has added value.

#### Characteristics of the social context

Medical professionals reported a *culture* within their work environment in which psychological health was acknowledged but often not openly discussed.

In both hospitals, psychologists, physicians, and nurses reported having well-functioning *cooperation* between disciplines characterized by low-threshold communication, consultation, and referral. This was facilitated by the visibility of psychologists at medical departments, symposia, and committees. A barrier in the *cooperation* was a lack of communication between different providers about patient care trajectories. Physicians had little insight into what happened to the patient after referral to the psychologist or primary care services.

Physician: Sometimes, it’s the way of communication. Systems that don’t connect with each other or transfer of information that doesn’t happen, issues with waiting lists. And sometimes, we don’t even know about each other. Or we don’t follow up with the patient, because we think he’s under the care of the psychologist now and I can let go of him for a while and will take over his care later on.

Lack of understanding about FCR was an important barrier in the *patient social environment*. Loved ones assumed that patients would be relieved and pick up their normal life again. FCR was also less visible than the physical consequences of disease and treatment. Patients missed genuine attention, which resulted in feelings of loneliness and increased the threshold to talk about FCR. Some patients also experienced a taboo on talking about psychological help which could made them hesitant to seek professional help. Social support related to FCR is currently addressed as a theme in one of the sessions of SWORD, which was a facilitator for the *patient social environment*. For some, it would be helpful if the environment even had a more active role, by following a partner program or joining treatment sessions.

Patient: People don’t understand it [FCR], even when you explain it. Because you should be happy, because it is [the cancer] is gone. [..] Yeah, at least, you are still alive. [..] Then I think to myself, that is right, but it [cancer] is still something you can die from and it could still recur.

The last important facilitator was the *patient-professional relation*. Participants reported that disclosure of FCR, willingness to seek help, and motivation to engage actively in treatment were more probable when they felt that they had a connection with their healthcare professional. Important aspects of the relationship were trust, openness, and feeling seen as a person.

#### Characteristics of the organization

An organizational barrier for implementation mentioned in both hospitals was the limited *capacity* of psychologists which led to several problems. Patients reported long waiting lists for psychological treatment and psychologists reported having full agenda, making it difficult to stick to the time schedule of the SWORD manual. Implementing a new treatment like SWORD might lead to an increase of referrals for FCR. Increasing capacity was a difficult process hindered by limited budget for psychological care. Patients in the POC also reported long waiting time but this was not explicitly mentioned in relation to limited capacity.

Manager: I have a fixed number of clinical staff, which always creates tension. If there is more clinical demand, I either cannot meet this demand or I can only meet the demand if we do less of something else [treating other patient groups].

Other important barriers in both hospital settings were related to the *organization of care processes* relevant for referral to SWORD. The structure and frequency of follow-up appointments, which are important moments to identify FCR, are mostly based on medical necessity. Therefore, some patients receive regular follow-up appointments while others receive none. Furthermore, FCR is insufficiently embedded within the infrastructure of consultation and referral. Psychological screening instruments such as the distress thermometer are poorly accessible and there is lack of guidelines on how FCR should be addressed, by whom, and how they should refer to psychological services. Altogether this makes current referral depend highly on individual professionals. Facilitators for referral to SWORD in both hospital settings included a well-established multidisciplinary consultation structure and the existence of case managers (often nurse specialists) that have frequent and close contact with patients.

Psychologist: So to say, our infrastructure for the detection of all sorts of psychological problems is very vulnerable at the moment and it falls short. That is the Achilles’ heel.

Organizational barriers related to *capacity for organizational change* include hospitals main focus being on medical health and consequently less priority for the implementation of SWORD than the POC. Furthermore, complex legal procedures for the approval of new (eHealth) innovations could slow down implementation. Within the POC, there was limited capacity to facilitate the training of psychologists within the mental healthcare reimbursement model. Facilitators included that eHealth was embedded within the organizations vision in the regional hospital and POC, and that the POC already had an active structure for implementation of innovations.

#### Characteristics of the economic and political context

A facilitator in related to *finances* is that SWORD fits within the existing financial structures of psycho-oncological care in the Netherlands. The length and the number of therapy sessions in SWORD do not exceed the therapy time that is covered by health insurances. In contrast, the costs of implementing and maintaining SWORD (e.g., keeping it up to date) are not covered by existing parties, and therefore warrant attention.

Another important *financial* barrier is the existence of multiple commercial eHealth platforms in the Netherlands. Institutions must pay multiple license fees when they want to use psychological interventions from different platforms, and these fees are not covered by health insurances. Implementing SWORD may be (financially) problematic in institutions that already have contracts with other eHealth providers.

Related to *policy*, conflicting opinions were found about in which settings SWORD should be implemented. Some argued that SWORD should be solely offered by psychologists in hospitals since these have knowledge to comprehend the relation between mental and medical health and distinguish normal from abnormal fears. Others argued that there is a growing group of cancer survivors, and in order to reach these patients, it is crucial that SWORD is available for psychologists in all settings. Some patients preferred treatment outside the hospital as this setting confronted them with the medical treatment and their patient role.

Implementation expert: Knowing how large the group [of patients] is who suffer from fear of recurrence, you must make sure you can meet the needs of these people.

Finally, there is no nationwide *infrastructure for the implementation* of evidence-based psychological interventions. The development of psychological interventions like SWORD is performed by research groups or clinicians within defined research projects. Developers often lack the time, finances, and expertise to invest in further implementation. Many of the barriers for implementation are common across psychological interventions, and therefore, it would be beneficial to solve these in a centralized manner.

Implementation expert: You want to avoid that every hospital and every research group has to reinvent the wheel or start new conversations about it. You really want to look at what the central issues are.

## Discussion

In this study, we identified barriers and facilitators for the implementation of an evidence-based blended psychological intervention for FCR (SWORD) within real-world psycho-oncological care among important stakeholders in three healthcare settings. Both patients and professionals emphasized the necessity of a psychological intervention specific for FCR. Overall, participants were positive about SWORD (innovation domain) and specifically mentioned its credibility, applicability, and relevance. Physicians and nurses (professional domain) reported lack of self-efficacy, knowledge, and skills to address and refer for FCR. SWORD matched psychologists knowledge and skills which was a facilitator, while a critical attitude towards protocolized treatments, cognitive behavioral therapy, or eHealth were barriers. Patient barriers included lack of FCR awareness, negative expectations of psychotherapy, and inability or unwillingness to actively engage in treatment. Social context barriers were poor communication between different healthcare professionals and lack of understanding about FCR within the patient social environment. Organizational barriers included inadequate referral structures to psychological services, limited capacity of psychologists, and legal procedures for new innovations. Lack of a national implementation structure for evidence-based psycho-oncological interventions and eHealth platform costs were barriers in the economic and political context domain. Barriers and facilitators in different domains often reflected the same problem from different angles.

Adequate detection of and referral for FCR are important preconditions for the success of FCR interventions [18]. Our results are in line with previous studies indicating that FCR is insufficiently addressed in medical consultations [17, 18, 22–24], and we found this to be a multidimensional barrier. Patients were often not aware of the availability of FCR interventions, had negative expectations of psychological help, or were reluctant to talk about mental health with their physician or nurse. For physicians and nurses, the most important barriers were lack of time and insufficient knowledge, skills, and self-efficacy to address FCR, and to differentiate between mild and adaptive FCR. In the social domain, referral was complicated by poor communication between different professionals and in the organization domain by inconsistent follow-up, lack of formal screening tools, and inadequate referral structures. Consequently, FCR might remain undetected in many cases, especially for patients with avoidant coping. To overcome these barriers, introducing FCR should not be based on patients’ initiative or professionals individual routines [23] but rather be a universal step for all patients within routine medical oncology follow-up [7, 25]. Sustainable integration of FCR screening requires a multidimensional approach including sufficient follow-up monitoring, clear guidelines on who should screen for FCR at what time [12, 23], the development and implementation of brief and feasible FCR screening tools [12, 17, 23, 24], the use of reminders [23], sufficient resources (e.g. time and staff) [18, 23], formal training of referrers [23], and interdisciplinary learning [23]. At the same time, strategies to improve FCR detection should be carefully evaluated in terms of feasibility and tailored to individual settings.

Doubt about when SWORD would be the most appropriate treatment was an important barrier for psychologists. While in study contexts FCR interventions are offered following strict in- and exclusion criteria, real-world clinical decision-making is a more complex process involving clinical assessment of the level of FCR and other factors such as comorbidity, patient background, capability, needs, and preferences. Psychologists could be assisted by training and by feasible decisional tools [7] such as guiding questions and responses indicating clinical FCR [26]. Furthermore, (contra) indications for FCR interventions should more specifically be determined. Special attention should be given to the suitability of psycho-oncology interventions including SWORD for patients who do not speak the dominant language, who are often underrepresented in (intervention) studies. FCR interventions should either be translated or made less language-dependent which may also improve accessibility for patients with low literacy. For patients, the most important precondition to start treatment was motivation to actively engage in the treatment, which was in turn largely related to the time at which that SWORD was offered. For most patients, interventions for high FCR are optimally offered after the challenging period of medical treatment and the first months of physical, psychological. and social (re)adaptation. This is consistent with the rationale of SWORD [13], which is offered typically not earlier than 6 months post treatment. An earlier timing might be appropriate for low-intensity interventions that may prevent high FCR [27]. An important question for future research is whether there is a most optimal timing to offer different types of FCR interventions or whether this should be assessed from person to person.

Most evidence-based psychological treatments involve treatment protocols or manuals, providing guidance on the content, structure, and length. In our study, most psychologists’ current routines did not involve treatment protocols. They were concerned that protocols would not do right to patients’ individual needs and would not fit with patients with complex problems. In accordance with a study investigating barriers and facilitators for the sustainable delivery of the FCR intervention ConquerFear [18], some psychologists preferred to use protocols in a flexible way, e.g., by omitting, adding, and tailoring elements or modifying structure and length of sessions. Mixed feelings regarding the use of protocols were also reported by patients in our study, with some having concerns that it would not match their personal needs, while others perceived the protocol as guidance that provided clarity about the process and trust in the outcome. When considering flexibility, it is important to distinguish between modifications that do not significantly alter active ingredients and modifications that do alter active ingredients of the treatment and consequently may reduce treatment effectivity [28]. As little had been published about the mechanisms of action of FCR interventions, and most FCR interventions have not been evaluated in a real-world setting, it is not known how flexible delivery would affect their effectivity in reducing FCR. These questions might be important to answer in future research [12]. At the same time, psychologists should be educated about and equipped for using intervention manuals as a guide within the therapeutic relationship, rather than a directive. Concerns about protocols could be reduced by training and supervision [28], an organizational culture supporting the use of guided treatments [29], and working with dedicated local psychologists that advocate the complementary benefits of guided treatments, also referred to as champions [2]. Furthermore, we suggest to refer to these treatments as guideline, as the word protocol might implicate that it is a directive.

In the context of a broader discussion about the most appropriate place to treat FCR, we conducted this study within three settings: an academic hospital, a general hospital, and a POC. In terms of length, content, and structure, SWORD fitted well within the current care at all settings, which is an important facilitator for implementation [18]. We found several differences between the academic and general hospitals when it came to current care or barriers. In contrast to the POC, the hospitals had a higher priority on physical care compared to the POC which might slow down implementation. Also, the accessibility of hospitals might be a problem since there is limited capacity of psychologists and the medical healthcare reimbursement limits the number of patients that can be treated. In the POC, there was less room for flexibility within the mental healthcare reimbursement models. However, this service had a stronger existing infrastructure when it came to implementation of innovations and sustainable integration of eHealth. The opinion of the participants about the most appropriate place to implement FCR interventions varied, with expertise (perceived as higher in hospital settings) and accessibility (perceived as better outside the hospital setting) as the most important trade-off. POC’s and other primary mental healthcare settings might indeed be better accessible in terms of capacity and proximity and because of the non-medical environment. However, psycho-oncological care in these settings is not always covered, as the mental healthcare reimbursement requires a formal DSM diagnosis, and this is not always applicable for patients with FCR or other psycho-oncological problems. Evaluation of the costs, feasibility, and effectivity of FCR interventions in different settings might contribute to this discussion.

As blended care is a relatively new development in the last decades, it was not surprising that important barriers and facilitators were related to the eHealth component of SWORD. Most professionals were open to eHealth and reported benefits including improved efficiency, information transfer, and accessibility. Psychologists expected eHealth to be a barrier for patients, but interestingly, patients themselves were open to blended care and experienced sufficient eHealth literacy, which are important prerequisites for adherence [30]. This result is consistent with previous literature suggesting that patients are likely to accept and adhere to blended care which combines the advantages of online exercises with offline psychological guidance [30, 31]. Some patients did prefer offline assignments, but blended treatments can be tailored, making only few people fully unfit for blended care [30]. The most important challenges regarding eHealth were practical, including its integration within existing digital systems and financing. License fees are high due to the distribution of psychological interventions across different commercial eHealth platforms, and these fees are often not covered by healthcare reimbursement models. As a result, many evidence-based eHealth interventions for cancer survivors developed in the Netherlands are not yet available in clinical practice.

Few studies up to date have looked at the broader societal and economic context of implementing psycho-oncological care. A crucial barrier found in this study was the lack of a central organizational and funding structure that ensures implementation of evidence-based psychological interventions. As emphasized through the previous sections, implementation of FCR interventions requires a multidimensional strategy and involves system changes rather than individual behavior change [32]. This is rarely achieved by individual studies or researchers initiatives which are bound to limited research project funding [33] and often do not have the expertise, credibility, and contacts to ensure implementation [34]. We therefore stress the importance of interdisciplinary collaboration and a centralized approach towards implementation of psycho-oncological innovations.

Our study was conducted following a well-known implementation framework and data was collected and analyzed in a systematic and rigorous way. A key strength of our study is that we assessed these barriers across multiple domains and with all important stakeholders while previous studies mostly focused on either medical staff or psychologists. This increases the credibility of the results and does right to the multidimensionality of improving FCR care within complex eco-systems of psycho-oncology enabling durable implementation [32]. Our study also knows some limitations. First, participants had no experience with SWORD, and therefore, the results identify expected barriers and facilitators rather than observed factors [23]. Second, professionals were selected by a psychologist within their setting, which possibly created a bias of referrers that were more open to mental health. Third, the majority of patient participants had breast cancer. However, between these participants with breast cancer, there was important diversity in other characteristics (i.e., setting, age, and level of FCR). Finally, we identified barriers specific for SWORD and within the Netherlands, possibly limiting generalizability. Specifically, the mandatory healthcare insurance in the Netherlands might make psycho-oncological care better accessible relative to other countries, although in some settings reimbursement is not guaranteed and additional expenses are needed. The organization of care processes, access to healthcare, and openness to mental health may also differ between countries. At the same time, many barriers and facilitators that we identified in the innovation, patient, professional, and organization domains have been reported in studies about implementation of psycho-oncological care in other countries [7, 17, 18], suggesting that, while psycho-oncological care is highly diverse across countries, some important determinants for implementation might be universal.

### Conclusion and recommendation

This interview study assessed barriers and facilitators for successful implementation of an evidence-based blended psycho-oncological intervention for FCR in real-world care. Facilitators of SWORD included its high reliability, accessibility, and relevance. Important barriers were insufficient screening and referral to psychological services, affected by patient, professional, social, and organizational factors; lack of consensus regarding treatment indication for SWORD; practical and financial challenges related to the eHealth platform; organizations capacity for implementation of new innovations; and lack of a national structure for the implementation of evidence-based psychological interventions. Barriers and facilitators identified in this study will inform tailored implementation strategies for SWORD and are valuable for the implementation of other psycho-oncological interventions. Recommendations for researchers, policy makers, and professionals involved in the implementation of psycho-oncological interventions are listed in Box 2.

Box 2 Recommendations for translating evidence-based psycho-oncological interventions into routine care.

**Table:** 

**-**To guide implementation, it is crucial to understand the real-world care context in which the intervention will be implemented, as this is often different from the setting in which it was tested
**-**While previous studies predominantly collected healthcare providers views, the perspectives of patients, business managers, policy advisers, and reimbursements experts are equally important as they have different roles during implementation
-Determinant frameworks can serve as theoretical guide to systematically identify barriers and facilitators in different domains to develop a tailored implementation strategy

## Supplementary Information

Below is the link to the electronic supplementary material.

Supplementary file1 (DOCX 15 KB)
