# Supplementary material for: Barriers and facilitators for implementation of the SWORD evidence-based psychological intervention for fear of cancer recurrence in three different healthcare settings
Source: J Cancer Surviv. 2022 Nov 4;17(4):1057–71. doi: 10.1007/s11764-022-01285-x (PMC9638257; doi:10.1007/s11764-022-01285-x)
Supplement: Supplementary file 1 — Supplementary file1 (DOCX 15 KB) [file 11764_2022_1285_MOESM1_ESM.docx]

**Supplementary file 1. Topic guides for semi-structured interviews with patients and professionals**

**1. Topic guide patients**

Start of the interview:

- Introduction of the interviewer
- Introduction to the FORwards project
- Introduction to SWORD: verbal explanation of intervention background, goal, structure and content; screenshots of the eHealth environment; exemplary exercises

Topic 1: Personal experiences with FCR

- personal experiences with FCR
- need for help with FCR
- received care/support for FCR

Topic 2: General impression of SWORD

Topic 3: Barriers and facilitators for implementation

- characteristics of the innovation
- characteristics of the patient
- characteristics of the professionals
- characteristics of the social context
- characteristics of the organization
- characteristics of the economic and political context

End of the interview

- Interviewer provides summary and asks for additional information
- End recording, thanking for participation

**2. Topic guide professionals**

Start of the interview:

- Introduction of the interviewer
- Introduction to the FORwards project
- Introduction to SWORD: verbal explanation of intervention background, goal, structure and content; screenshots of the eHealth environment; exemplary exercises

Topic 1: Current care for FCR

Topic 2: General impression of SWORD

Topic 3: Barriers and facilitators for implementation

- characteristics of the innovation
- characteristics of the patient
- characteristics of the professionals
- characteristics of the social context
- characteristics of the organization
- characteristics of the economic and political context

End of the interview

- Interviewer provides summary and asks for additional information
- End recording, thanking for participation
